# Source Evaluation and Trace Metal Contamination in Benthic Sediments from Equatorial Ecosystems Using Multivariate Statistical Techniques

**DOI:** 10.1371/journal.pone.0156485

**Published:** 2016-06-03

**Authors:** Nsikak U. Benson, Francis E. Asuquo, Akan B. Williams, Joseph P. Essien, Cyril I. Ekong, Otobong Akpabio, Abaas A. Olajire

**Affiliations:** 1 Environmental Chemistry Unit, Department of Chemistry, Covenant University, Ota, Nigeria; 2 Faculty of Marine Sciences, University of Calabar, Calabar, Nigeria; 3 Department of Microbiology, University of Uyo, Uyo, Nigeria; 4 Department of Chemical Science, Ritman University, Ikot Ekpene, Akwa Ibom State, Nigeria; 5 Department of Chemistry, University of Uyo, Uyo, Akwa Ibom State, Nigeria; 6 Industrial and Environmental Chemistry Unit, Department of Pure and Applied Chemistry, Ladoke Akintola, University of Technology, Ogbomoso, Nigeria; University of Vigo, SPAIN

## Abstract

Trace metals (Cd, Cr, Cu, Ni and Pb) concentrations in benthic sediments were analyzed through multi-step fractionation scheme to assess the levels and sources of contamination in estuarine, riverine and freshwater ecosystems in Niger Delta (Nigeria). The degree of contamination was assessed using the individual contamination factors (ICF) and global contamination factor (GCF). Multivariate statistical approaches including principal component analysis (PCA), cluster analysis and correlation test were employed to evaluate the interrelationships and associated sources of contamination. The spatial distribution of metal concentrations followed the pattern Pb>Cu>Cr>Cd>Ni. Ecological risk index by ICF showed significant potential mobility and bioavailability for Cu, Cu and Ni. The ICF contamination trend in the benthic sediments at all studied sites was Cu>Cr>Ni>Cd>Pb. The principal component and agglomerative clustering analyses indicate that trace metals contamination in the ecosystems was influenced by multiple pollution sources.

## 1. Introduction

The rapid industrial and socioeconomic development of the Niger Delta of Nigeria has resulted in increasing heavy metal pollution of coastal aquatic ecosystems (tidal estuaries and creeks, rivers, mixohaline lagoons and mangrove swamps) located in the region [1–4]. Most of the equatorial water systems in the Niger Delta serve as primary recipients of petroleum exploration-exploitation wastes, onshore and offshore industrial sewage, chemical contaminants, domestic and office wastes generated by multinational oil companies that are found in the region. In addition, these aquatic ecosystems have also witnessed the uncontrolled use of banned chemicals by local fishermen for fishing purposes. Several offshore and onshore incidents of crude oil spillage into water bodies in the region have been reported [4–6]. Studies have indicated enhanced levels of petroleum hydrocarbons and trace metals in surface water, sediments and biota associated with oil pollution [3, 6–8].

Trace metals contamination in aquatic ecosystems constitutes significant health and environmental hazard for fishes, humans, invertebrates and mangroves. The relative magnitude of occurrence, persistence, varied sources, bioaccumulative nature, and toxicity of trace metals in coastal aquatic ecosystems are increasingly receiving global attention [9–14]. Metals are introduced into aquatic systems through natural processes such as rock weathering and anthropogenic inputs such as agricultural activities, mining, atmospheric depositions, domestic and industrial wastewater/effluents, municipal wastes, soil erosion and runoff and domestic sewage [15–17]. Once released into aquatic ecosystems, trace metals are primarily associated with sediments and related sedimentary particulate matter. Therefore, sediments are critical sinks or sources of pollutants [18–21]. Coastal estuarine and riverine sediments are critical components of these fragile ecosystems and are particularly prone to contamination [2–4, 21–24].

Multivariate statistical approaches have been widely employed in identifying the natural and/or anthropogenic sources of metal contamination in estuarine/marine water and sediments [25–31]. Statistical methods commonly used include the principal component analysis (PCA), cluster analysis, and correlation test. The principal component analysis is a statistical tool frequently employed for the elucidation of information of multivariable origin by simplifying statistics, which means employing less comprehensive indices instead of more relative indices to separate natural and anthropogenic trace metals influence [32, 33, 34]. PCA provides simple visualization of the interrelationships that exists among quantitative observations/variables in complex or large datasets including data of different distributions in the sediment fractions [35, 36]. It has been applied to interpret the variations in sedimentary trace metals and organic matter concentrations as well as assist in analyzing the origin of metal contamination with the spatial distribution of trace elements [27]. The agglomerative hierarchical clustering (AHC) analysis provides a measure of similarity in assessing the associations among the observed variables and the sampling sites, while correlation analysis presents a statistical characterization of a quantitative variable by other quantitative variables to establish their relationships using the correlation coefficient.

The objectives of this study are to: (i) determine the spatio-temporal distributions and fractionations of five trace metals (Cd, Cr, Cu, Ni and Pb) in benthic sediments; (ii) evaluate the anthropogenic and/or natural inputs of trace metals using multivariate statistical approaches; and (iii) explore the interrelationships among the trace metals and sediment constituents.

## 2.0 Materials and Method

### 2.1 Study area

**Fig 1:**
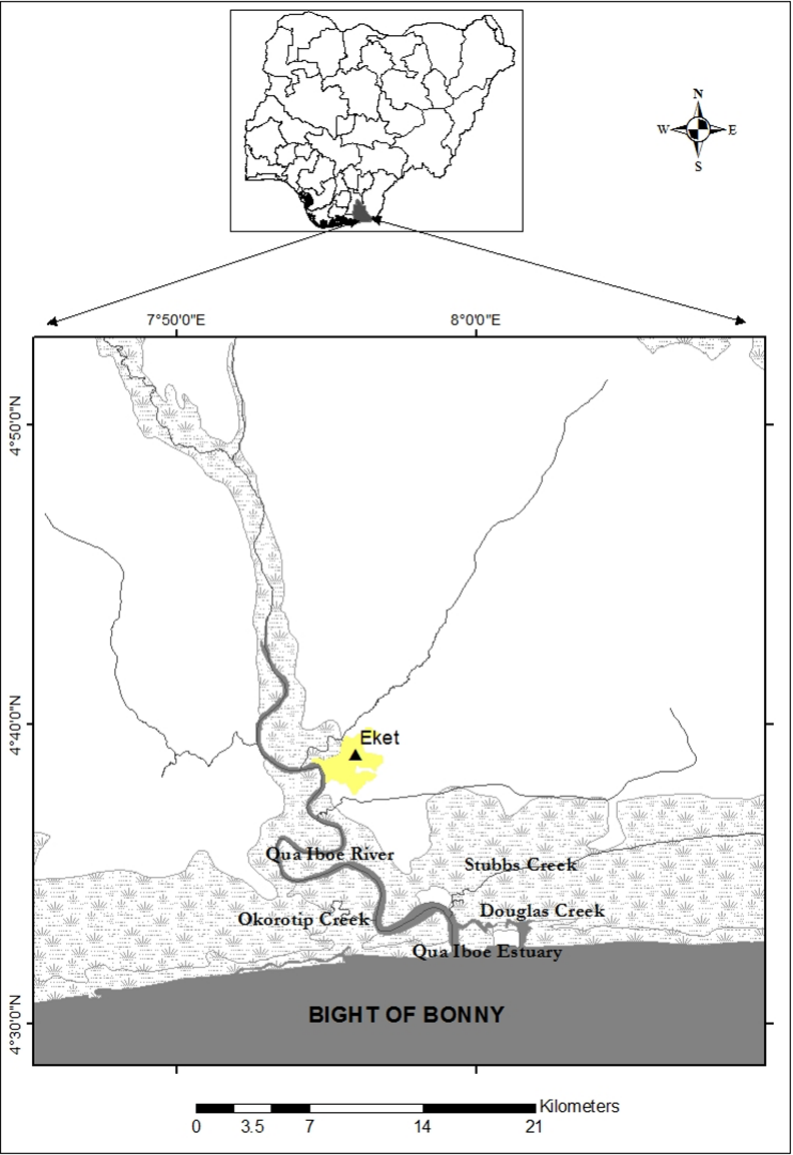
Map of study area showing the ecosystems in Niger Delta region of Nigeria (Inset: Map of Nigeria).

Fig 1 shows the map of the investigated freshwater, riverine and estuarine ecosystems in this study. The aquatic ecosystems considered in this study are Douglas Creek (DOU) (4.55°S, 8.00°N), Okorotip Creek (OKT) (4.56°S, 7.93°N), Stubbs Creek (STB) (4.60°S, 7.99°N), Qua Iboe Estuary (QUE) (4.53°S, 7.99°N) and Qua Iboe River (QUR) (4.58°S, 7.93°N).

### 2.2 Sediment sampling and analyses

#### 2.2.1 Collection and pretreatment of sediment samples

Five (5) sampling sites were mapped and designated for sediment samples collection. Triplicate sediment samples from each designated sites were collected monthly using a modified van Veen (0.1 m^2^) grab sampler and were preserved in clean, well-labeled glass bottles. In all, fifteen sediment samples were collected each month from five sampling sites during the wet season (June, July August) resulting in forty-five benthic sediment samples for the period. A similar routine was repeated for the dry season months (November, December and January). Therefore, ninety (90) benthic sediment samples were collected from the study locations during this investigation. After collection, all samples were stored in ice-packed coolers and transported to the laboratory. These samples were further treated by refrigeration in the laboratory at 4^°^C to inactivate microbes and preserve the integrity of the samples prior to analysis. In the laboratory, the sediment samples were dried in an oven maintained at 105±0.5°C, homogenized, comminuted using a hand mortar and sieved using a 2 mm mesh sieve prior to leaching [37]. Coning and quartering method was used to obtain subsamples from the respective composite samples.

#### 2.2.2 Sediment characterization analysis

The major sediments constituents considered in this study included total organic carbon, silt, clay and sand. The preparation, extraction and quantitation of benthic sediment samples for the determination of total organic carbon (TOC) followed the wet chemistry technique as described by [38]. Fine-grained portion of grain-size distributions were performed by sedimentation method [39, 40].

#### 2.2.3 Chemical fractionation of sediment and elemental analysis

For the purpose of classifying the bioavailable metallic species in each sample, five sequential chemical extractions were performed with the objective of identifying the metal classifications influenced by various environmental conditions: (a) exchangeable, (b) bound to carbonates, (c) bound to iron and manganese oxides (reducible), (d) bound to organic matter (oxidizable), and (e) residual [41]. The selective extraction of fraction A was performed using 1.0 g of sieved sediment at room temperature for one hour with 8.0 mL of 1 M MgCl_2_ solution at pH 7.0 with continuous agitation. This fraction sometimes known as acid-soluble fraction provides information on the capacity of the sediment to absorb and desorp trace metals in relation to changes in the ionic composition of the sediment. Sediment residues from fraction A were leached at room temperature with 8.0 mL of 1 M sodium acetate at a pH of 5.0 (adjusted using acetic acid) with continuous agitation to obtain metals that are associated with carbonates (fraction B). For the reducible fraction (fraction C) extraction, sediment residues obtained from fraction B were extracted with 20 mL of 0.04 M hydroxyl ammonium chloride in 25% (v/v) acetic acid for 6 hr at 96°C with occasional agitation of the solution. Fraction C constitutes trace metals associated with iron and manganese oxides and is sensitive to redox potential variations.

In order to obtain trace metals that are associated with organic matter (oxidisable) (fraction D), sediment residue in fraction C were extracted with 3.0 mL of 0.02 M nitric acid and 5.0 mL of 30% hydrogen peroxide solution and heated for 2 hr at 85°C with intermittent agitation. Thereafter, 3.0 mL of 30% hydrogen peroxide was added and pH of mixture adjusted to 2.0 using nitric acid, and heated at 85°C for 3 hr with continuous agitation. On cooling, 5.0 mL of 3.2 M ammonium acetate in 20% (v/v) nitric was added and the mixture diluted to 20.0 mL with continuous agitation for 30 min. This fraction gave trace metals bound to variable forms of organic matter that can be released under oxidizing conditions. Finally, fraction E (trace metals bound to the residue) were obtained through extraction of residue fraction from D in a Teflon vessel with a mixture of 50 mL of 40% hydrofluoric acid and 75 mL of 60% perchloric acid. The mixture was evaporated to dryness and 2.0 mL of 60% perchloric acid added and evaporated until white fumes were produced. Resultant residue was digested in 50 mL of 3 M hydrochloric acid. This fraction includes trace metals that are structurally bound to silicates. A summary of the Tessier sequential extraction procedure and the reagents used is presented in Table 1.

**Table 1:** Geochemical fractionation scheme of trace metals adopted for present study.

Extraction step	Fractionation phase	Nominal target phase	Reagents
Step 1	Fraction A	Exchangeable metals	MgCl_2_ (1.0 mol/dm^3^)
Step 2	Fraction B	Carbonates bound	NaOAc (1.0 mol/dm^3^) at pH = 5.0
Step 3	Fraction C	Oxides Fe/Mn	NH_2_OH.HCl (0.04 mol/dm^3^) / CH_3_COOH (4.4 mol/dm^3^)
Step 4	Fraction D	Organic matter and sulfides	HNO_3_ (0.02 mol/dm^3^) / H_2_O_2_ (12.8 mol/dm^3^); then NH_4_OAc (3.2 mol/dm^3^) at pH = 2.0
Step 5	Fraction E	Residual bound to silicates	HF/HClO_4_; then HCl (3.0 mol/dm^3^)

After extractions, the concentrations of cadmium (Cd), copper (Cu), chromium (Cr), lead (Pb) and nickel (Ni) in fractions A–E were determined using inductively coupled plasma spectrophotometer (ICP-AES). The detection limits were 0.02, 0.01, 0.02, 0.02 and 0.01 ppm for Cd, Cr, Cu, Pb and Ni respectively.

#### 2.2.4 Preparation of standard

In order to reduce the detrimental effects of overlapping spectral interferences on element quantitation during metal analyses, an interelement correction standard was prepared by using standardized solution of metals ions prepared from their salts. A mixture of commercially available 100 ppm stock solutions (BDH Grade) of Cd^2^, Cr^3^, Cu^2^, Pb^2^ and Ni^2^ were prepared as interelement working standard solutions to verify that the overlapping lines do not cause the detection of elements at concentrations above methods detection limits (MDLs) [42].

### 2.3 Statistical analysis and GIS

The analytical data obtained were analyzed using the XLSTAT-Pro software (AddinSoft, Inc., NY, USA). Pearson’s correlation analysis and Principal component analysis (PCA) were employed to explore the interrelationship among trace metals and between TOC and trace metals concentrations in sediment samples to identify their hypothetical source. The adequacy of data for PCA was confirmed with the Kaiser–Meyer–Olkin (KMO) and Bartlett’s sphericity tests. A difference of *p* < 0.05 was considered to be significant. Comparative and continuous summary descriptives were also performed. Also, the agglomerative hierarchical clustering (AHC) analysis was conducted on the observed dataset using Ward’s method [43], with Euclidean distances (proximity matrix) as a measure of similarity to assess the associations among the parameter and sampling sites. The AHC is an iterative classification method that initiates clustering by calculating the dissimilarity between different group of objects, and then two objects which when clustered together minimize a given agglomeration criterion are clustered together [44–46]. The software employed for mapping the spatial variations and risk assessment code index was ArcGIS Version 10.2 developed by ESRI. On the basis of the observed data, a linear combination of the observed values with weights was used to estimate the values of attributes at unsampled sites within the investigated aquatic ecosystems, which considers the direction of variations and incorporates trends into the interpolation to create better predictions. For this study, ordinary Kriging was adopted for estimation of geospatial data as:

$$Z^{*}(x_{0})=\sum_{i=1}^{n}\lambda_{i}Z(x_{i})$$ (1)

where Z*(*x*_0_) is the estimated values of Z a point *x*_0_, *Z*(*x*_*i*_) is the observed value at the point *x*_*i*_ and *λ*_*i*_ is the weight placed on *Z*(*x*_*i*_) [47].

### 2.4 Contamination assessments

#### 2.4.1 Individual contamination factor and global contamination factor

The degree of ecological risk associated with trace metals could be evaluated as a function their retention by determining the contamination factors of metals [48]. According to [49], a high contamination factor of the metals implies low retention time and high degree of risk to the aquatic environment. In this study, the individual contamination factors (ICF) and the global contamination factor (GCF) were computed for the investigated sampling sites during the wet and dry seasons. The ICF for the DOU, OKT, STB, QUE and QUR sites were calculated by dividing the sum of the non-residual fractionation concentrations (i.e. exchangeable, carbonates bound, acid-reducible and oxidizable-organic fractions) by the residual fraction for each site. The GCF for each investigated site was computed through the summation of the ICF for Cd, Cr, Cu, Ni and Pb for each site [36, 50]. The individual contamination factor and global contamination factor classifications are: ICF < 0 & GCF < 6 –indicates low, 1 < ICF < 3 & 6 < GCF < 12– moderate, 3 < ICF < 6 & 12 < GC < 24 –considerable and ICF > 6 & GCF > 24 –high contamination. The ICF and GCF were computed for each sampling site according to the following equations:

$$ICF_{m}=\frac{\sum_{i=A}^{D}C_{F_{i}}}{C_{F_{E}}}$$ (2)

$$GCF=\sum_{m=1}^{n}ICF_{m}$$ (3)

where $C_{F_{i}}$ denoted the concentration of each non-residual fraction and $C_{F_{E}}$ is the concentration of the residual fraction.

## 3.0 Results and Discussion

### 3.1. Trace metals content and characterization of sediment

**Fig 2:**
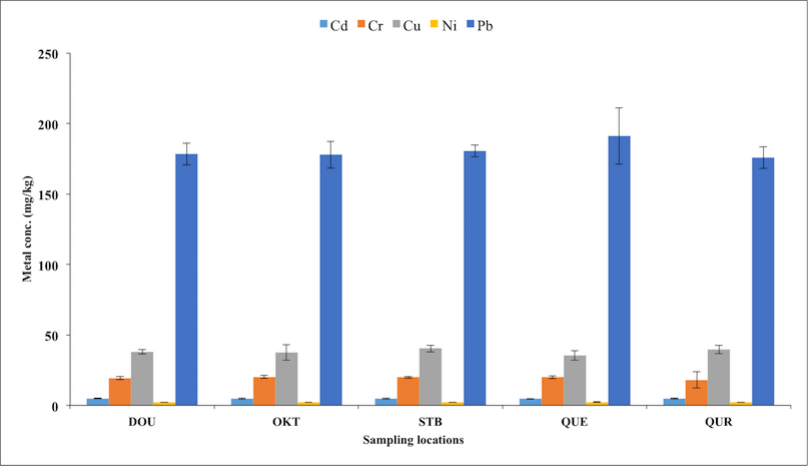
Trace metals concentrations (mg/kg) in sediment samples obtained for wet and dry seasons.

**Fig 3:**
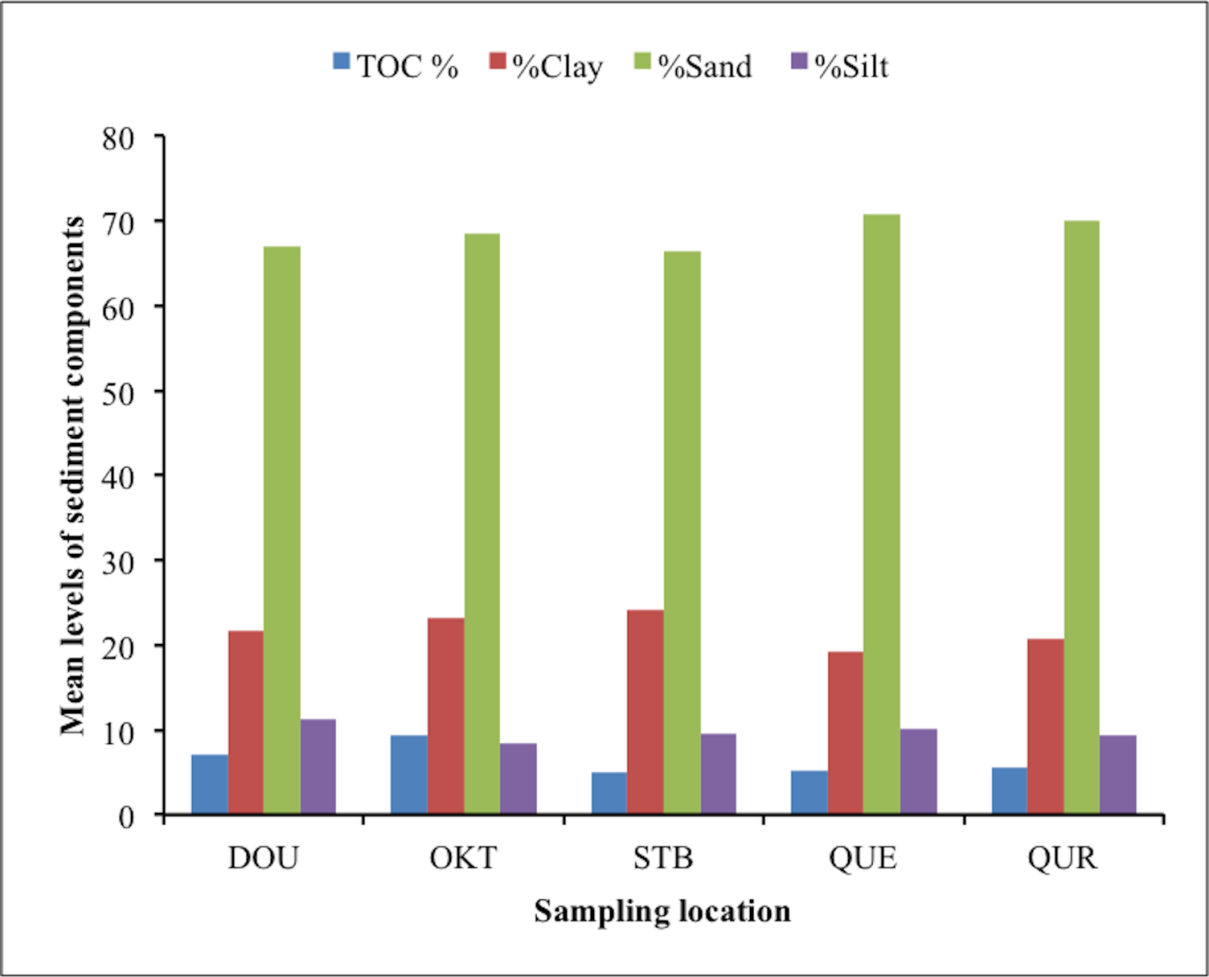
Sediment characterization variables in investigated ecosystems in Niger Delta.

**Fig 4:**
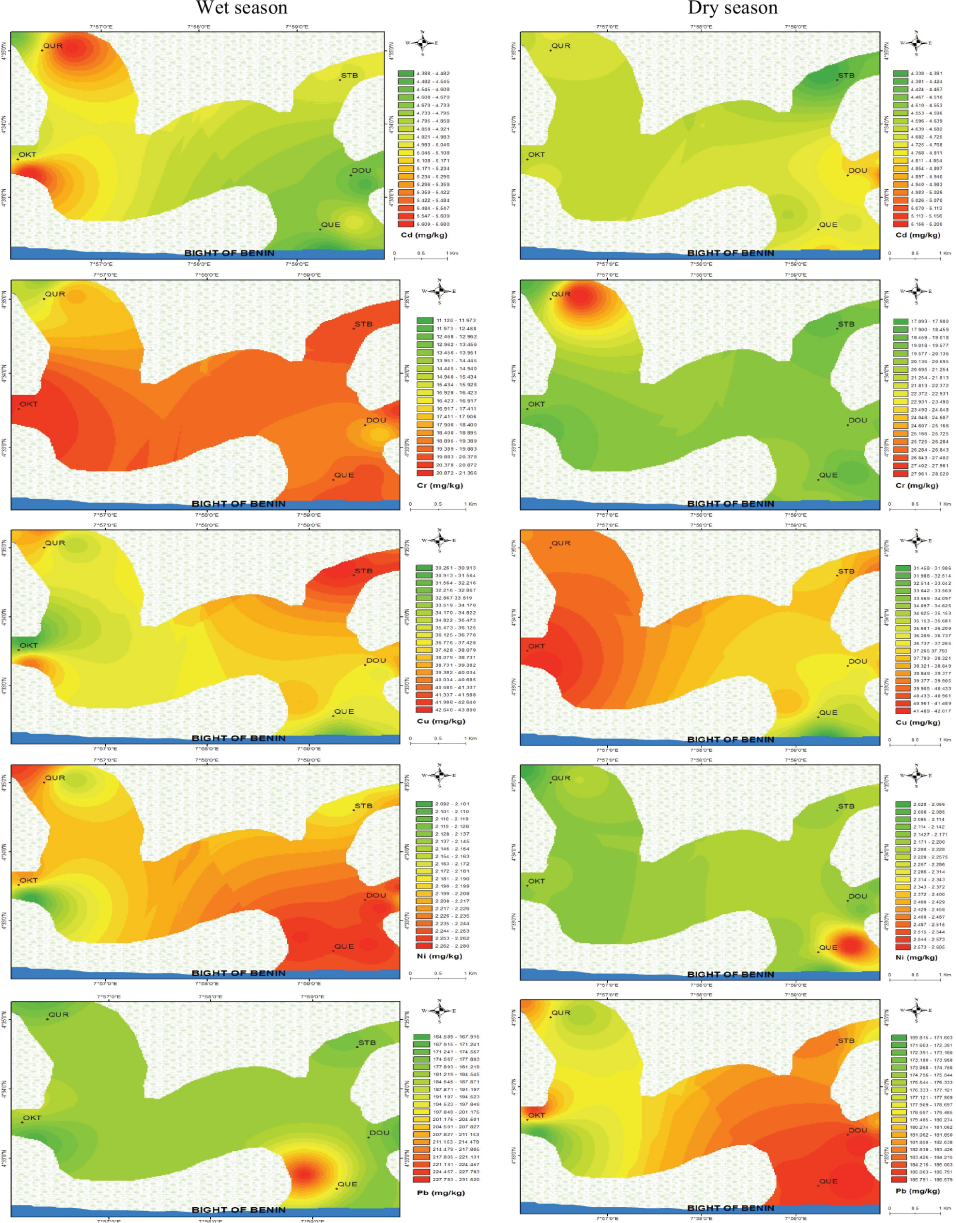
Spatial distribution of trace metals in benthic sediments of mangrove ecosystems of Niger Delta during wet and dry seasons.

The levels of trace metals (Cd, Cr, Cu, Ni, Pb), total organic carbon (TOC) content, silt, clay and sand in benthic sediments at various sites are presented in Fig 2 and Fig 3, while the spatial distribution of trace metals in the five studied ecosystems during the wet and dry seasons are shown in Fig 4. The concentrations of each studied trace metal indicated significant spatial variations that were characteristically distinctive and correlative with proximity to anthropogenic activities (industrial and domestic), nearshore area, fishing settlements, and shipping channel. Higher concentrations were observed more frequently near the Atlantic coast of the estuarine ecosystem resulting in comparable downstream concentration values especially at locations with intense anthropogenic activities. The concentrations of trace metals generally followed the pattern Pb>Cu>Cr>Cd>Ni.

The TOC values ranged from 5.55–10.77, 5.91–11.33, 6.82–11.23, 4.99–6.44, and 5.23 to 14.56% with mean values of 7.05±2.0, 8.46±2.11, 9.45±1.48, 5.24±0.71 and 9.34±3.59% at DOU, OKT, STB, QUE and QUR sites, respectively. The results indicate high organic contents in investigated sediment, implying high sedimentary metal affinity for humic substances, which might decrease trace metal bioavailability through complexing [31]. The silt content of sediments ranged from the lowest value of 8.64 at QUE site to 75.23% at QUR site with the highest mean value of 69.96±3.59%.

### 3.2 Trace metals pollution sources apportionment

The Pearson correlation coefficients for trace metals, total organic content, sand, clay and silt in asphyxiated benthic sediments from equatorial estuarine and freshwater ecosystems in Niger Delta are presented in the Pearson matrixes below (Table 2).

**Table 2:** Correlation matrix for trace metals, TOC, clay, sand and silt in benthic sediments.

		Cd	Cr	Cu	Ni	Pb	TOC%	%Clay	%Sand	%Silt
	Cd	**1**								
	Cr	0.070	**1**							
Douglas Creek	Cu	0.556	0.088	**1**						
	Ni	0.272	0.278	-0.013	**1**					
	Pb	0.299	0.056	-0.615	0.196	**1**				
	TOC%	-0.636	-0.600	-0.451	-0.774	-0.070	**1**			
	%Clay	0.646	0.643	-0.002	0.533	0.644	-0.761	**1**		
	%Sand	-0.477	-0.700	0.159	-0.288	-0.704	0.550	**-0.944**	**1**	
	%Silt	-0.758	-0.419	-0.218	-0.773	-0.436	**0.909**	**-0.887**	0.684	**1**
	Cd	**1**								
	Cr	-0.070	**1**							
Qua Iboe	Cu	0.202	-0.463	**1**						
Estuary	Ni	**0.849**	0.054	0.293	**1**					
	Pb	0.060	-0.566	0.540	-0.249	**1**				
	TOC%	-0.320	-0.535	0.194	-0.417	0.730	**1**			
	%Clay	0.466	-0.591	0.680	0.183	0.624	0.016	**1**		
	%Sand	-0.431	0.604	-0.655	-0.140	-0.613	-0.010	**-0.998**	**1**	
	%Silt	0.241	-0.635	0.505	-0.069	0.528	-0.016	**0.938**	**-0.957**	**1**
	Cd	**1**								
	Cr	0.232	**1**							
Okorotip Creek	Cu	0.231	-0.720	**1**						
	Ni	0.048	-0.168	0.312	**1**					
	Pb	-0.327	0.474	-0.676	-0.080	**1**				
	TOC %	0.645	0.100	0.269	-0.341	0.088	**1**			
	%Clay	0.333	-0.500	0.432	-0.544	-0.652	0.344	**1**		
	%Sand	0.692	-0.448	0.649	0.219	-0.321	0.652	0.460	**1**	
	%Silt	-0.671	0.522	-0.666	-0.017	0.466	-0.638	-0.683	**-0.963**	**1**
	Cd	**1**								
	Cr	0.464	**1**							
Stubbs Creek	Cu	0.761	0.361	**1**						
	Ni	0.596	-0.322	0.458	**1**					
	Pb	0.164	-0.148	-0.431	0.353	**1**				
	TOC %	-0.493	-0.436	-0.525	-0.043	-0.056	**1**			
	%Clay	**0.855**	0.526	0.653	0.207	-0.063	-0.436	**1**		
	%Sand	-0.689	-0.412	-0.497	-0.099	0.165	0.127	**-0.939**	**1**	
	%Silt	-0.010	-0.038	-0.086	-0.186	-0.310	0.614	0.348	-0.650	**1**
	Cd	**1**								
	Cr	-0.184	**1**							
Qua Iboe River	Cu	-0.451	-0.297	**1**						
	Ni	0.383	0.081	-0.011	**1**					
	Pb	0.139	0.455	-0.752	0.470	**1**				
	TOC %	0.784	-0.400	-0.573	0.436	0.405	**1**			
	%Clay	-0.743	0.037	0.559	0.293	0.074	-0.470	**1**		
	%Sand	0.579	0.683	-0.532	0.456	0.503	0.275	-0.439	**1**	
	%Silt	-0.373	-0.767	0.384	-0.610	-0.580	-0.134	0.124	**-0.946**	**1**

Values in bold are different from 0 with a significance level alpha = 0.05.

**Fig 5:**
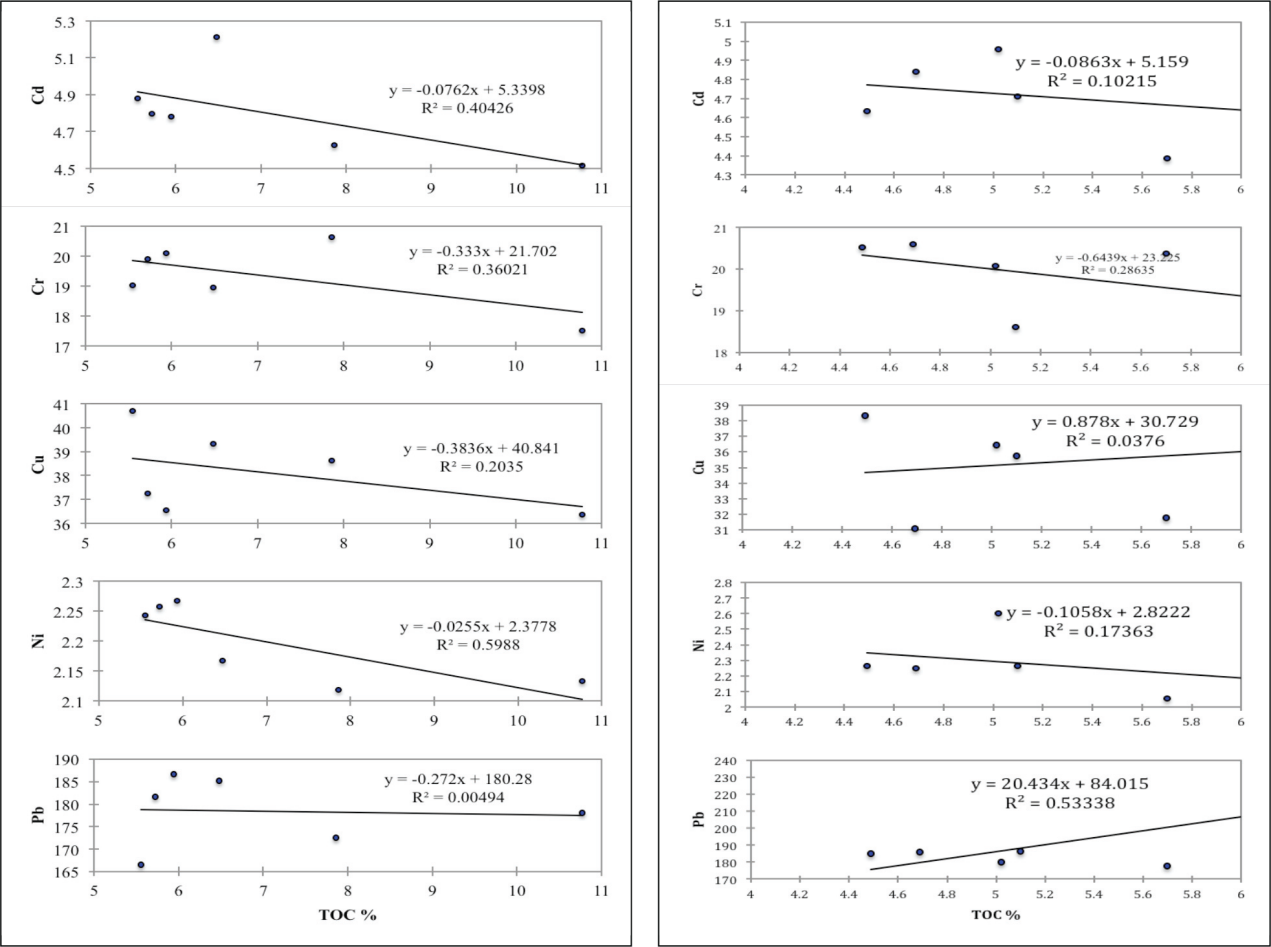
Scatter plots of trace metals and total organic contents in Douglas creek (left) and Qua Iboe estuary (right).

Pearson statistical analysis indicated weak but positive interelement relationships between Cr, Cu, Ni, Pb and Cd at DOU, STB and QUE sites. According to [51], strong but positive correlations between trace metals indicate that they might likely originate from common source(s), and possess mutual dependence and identical behavior during transportation between aquatic compartments. Cu and Cr showed negative correlations at OKT, QUE and QUR sites. In general, metal–metal interrelationships in the present study were either positive or negative but correlatively weak indicating the unlikelihood that most of the trace metals originated from the same source. Moreover, trace metals correlations with TOC, sand, silt and clay contents of sediments were mostly negative except at STB site where Cd showed a strong and significant relationship with clay. Cd mostly recorded positive correlations with clay at all sites except at QUR site. There was a negative correlation between TOC levels in sediment and trace metals at DOU and STB sites, while a mix of positive and negative correlations marked the interrelationships between TOC and metals at QUE, QUR and OKT. This probably suggests that different organic particulates and the physicochemical conditions of TOC might have been responsible for the transport and distribution of trace metals in these aquatic ecosystems. Representative scatter plots depicting the linear relationships between TOC% and trace metals in benthic sediment of the study sites are presented in Fig 5. The overall Kaiser-Meyer-Olkin (KMO) measure of sample adequacy coefficients were 0.31, 0.51, 0.50, 0.60 and 0.32 <1 for DOU, OKT, STB, QUE and QUR sites dataset, respectively, and the concomitant probability of Bartlett’s sphericity test was less than the significance level α = 0.05. Thus, the observations at OKT, STB and QUE were averagely adequate for a factor model [52].

#### 3.2.1 Principal component analysis

**Fig 6:**
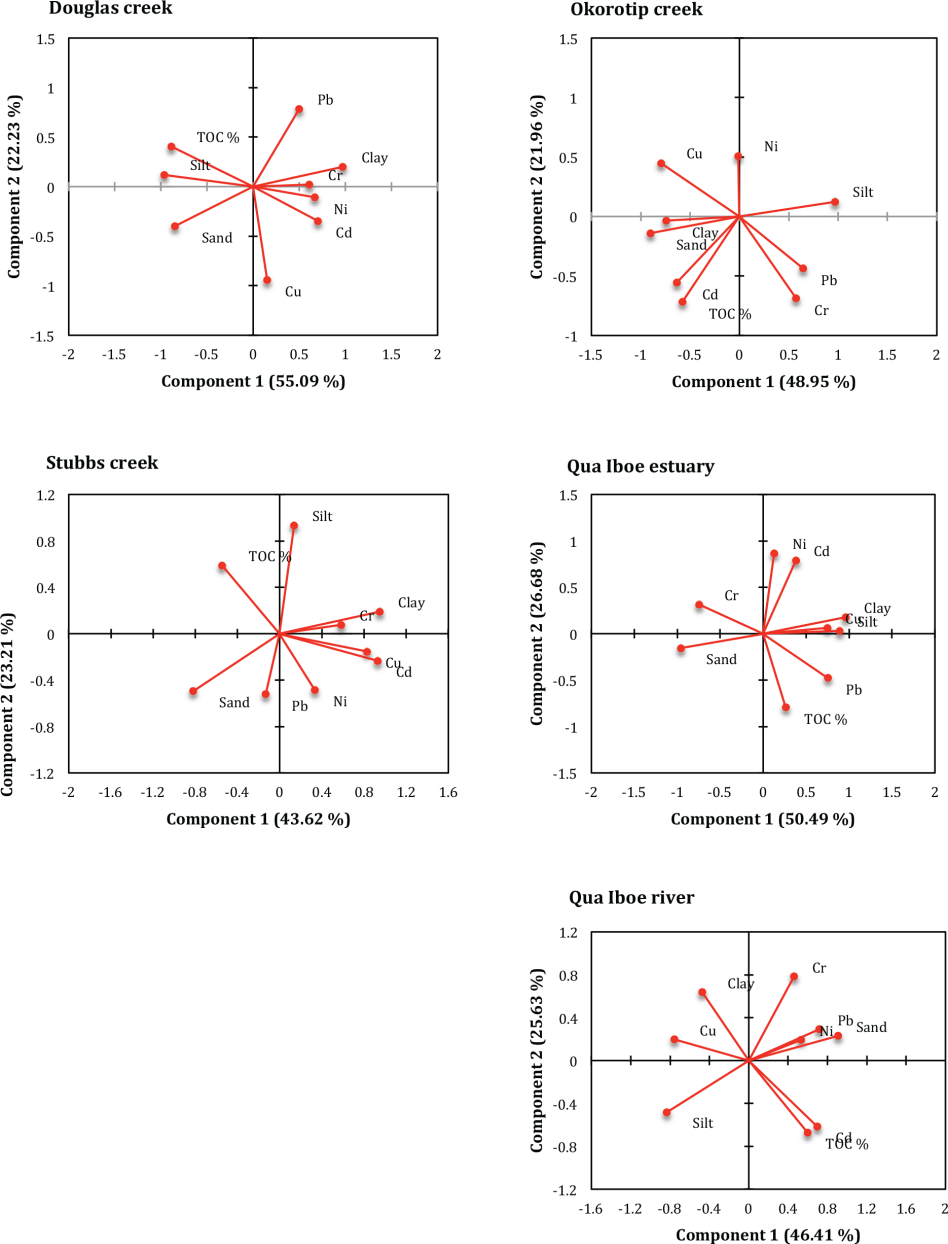
The two principal components calculated by PCA reflecting the relationship of investigated trace metals and sedimentary variables.

The rotated factor loadings of principal component analysis (PCA) conducted to evaluate the interrelationships of trace metals and the major constituents (clay, sand, silt and TOC) of the benthic sediments from the five studied aquatic ecosystems are presented in Table 3 and Fig 6. The different trace metals contamination behaviors were observed in all five studied ecosystems. As shown in Table 3, there were two principal components (PC1 and PC2) for sedimentary variables at the DOU, OKT, STB, QUE and QUR sites. Overall, the two principal components characterize 77.32, 70.91, 66.83, 77.17 and 72.05% of the accumulative contribution rate for the five trace metals and four sediment quantities at the DOU, OKT, STB, QUE and QUR sites, respectively. At the Douglas creek, the contribution of PC1 was 55.09% of the total variance, and had a positive load in the contribution of Cd, Ni and clay, indicating that these trace metals originated from anthropogenic activities such as industrial effluent or domestic sewage. Other sedimentary components such as sand, silt and TOC indicated significant negative relationships with PC1. The second principal component at DOU site contributed 22.23% of the total variance, and was only positively related to Cu and correlatively negative to Pb.

**Table 3:** Loadings of two principal components for benthic sedimentary variables.

	DOU		OKT		STB		QUE		QUR	
	PC1	PC2	PC1	PC2	PC1	PC2	PC1	PC2	PC1	PC2
Load of Cd	**0.703**	0.346	**-0.632**	0.556	**0.932**	-0.234	0.381	**0.791**	**0.698**	-0.618
Load of Cr	0.611	-0.018	0.574	**0.687**	0.586	0.075	**-0.746**	0.316	0.462	**0.784**
Load of Cu	0.152	**0.941**	**-0.794**	-0.448	**0.831**	-0.153	**0.747**	0.064	**-0.758**	0.198
Load of Ni	**0.669**	0.108	-0.012	-0.507	0.334	-0.486	0.128	**0.865**	0.534	0.195
Load of Pb	0.501	**-0.781**	**0.643**	0.435	-0.130	-0.524	**0.752**	-0.480	**0.717**	0.293
Load of Clay	**0.971**	-0.198	**-0.743**	0.036	**0.951**	0.188	**0.963**	0.180	-0.472	**0.638**
Load of Sand	**-0.845**	0.398	**-0.901**	0.140	**-0.821**	-0.496	**-0.958**	-0.155	**0.909**	0.229
Load of Silt	**-0.961**	-0.121	**0.968**	-0.123	0.138	**0.932**	**0.888**	0.028	**-0.833**	-0.483
Load of TOC	**-0.887**	-0.402	-0.577	**0.716**	-0.544	**0.588**	0.262	**-0.797**	0.599	**-0.674**
Eigenvalue	4.958	2.001	4.405	1.976	3.926	2.089	4.544	2.401	4.177	2.307
Contribution rate (%)	55.089	22.232	48.948	21.960	43.622	23.208	50.492	26.677	46.410	25.635
Cumulative %	55.089	77.322	48.948	70.908	43.622	66.830	50.492	77.168	46.410	72.045

Values in bold indicate strong loadings.

In the Okorotip creek, the first principal component, which accounted for 48.95% of the total variance was negatively related to the loads of Cd, Cu and major sediment constituents except silt. Silt was significantly positively related to PC1, while the load of Pb also indicated a positive relationship with PC1. The contribution rate of PC2 was 21.96% and related positively only with Cr and TOC, indicating a probable biogenic input. The correlatively high load of TOC indicated it as an important sedimentary metal ion conjugates.

In Table 3, PC1 accounted for 43.62% variability at STB site and was positively related to Cd, Cu and clay. This first principal component represents trace metals contamination from anthropogenic sources to sediments of the Stubbs creek. Sand was significantly negatively interrelated with PC1. The second principal component, which explained 23.21% of the total variance indicated significantly positive relationships with silt and TOC. In the Qua Iboe estuary, PC1 was positively related to Cu, Pb, clay and silt, and was also negatively related to Cr and sand. The contribution of the first principal component was 50.49%. The comparative significant loading of clay and silt with the first principal component elucidated the importance of fine-grained sediment minerals on the transport of trace metals within the estuarine ecosystem. It further highlighted the likelihood that Cd, Cu, Pb and Ni might have come from anthropogenic inputs. PC2, which explained 26.68% of the total variability, was significantly positively related to Cd and Ni. The total organic carbon content was negatively related to PC2.

Lastly, in the Qua Iboe River system, the contribution of the first principal component was 46.41% of the total variance. PC1 related positively with Cd, Pb and sand, indicating the influence of sand in capturing and distributing Cd and Pb ions in sediments of the Qua Iboe River. Also significantly negative relationships were established by PC1 for Cu and silt. PC2, which explained 25.64% variability, was positively related to Cu and clay, while indicating a negative interrelationship with organic carbon content.

#### 3.2.2 Agglomerative hierarchical clustering

**Fig 7:**
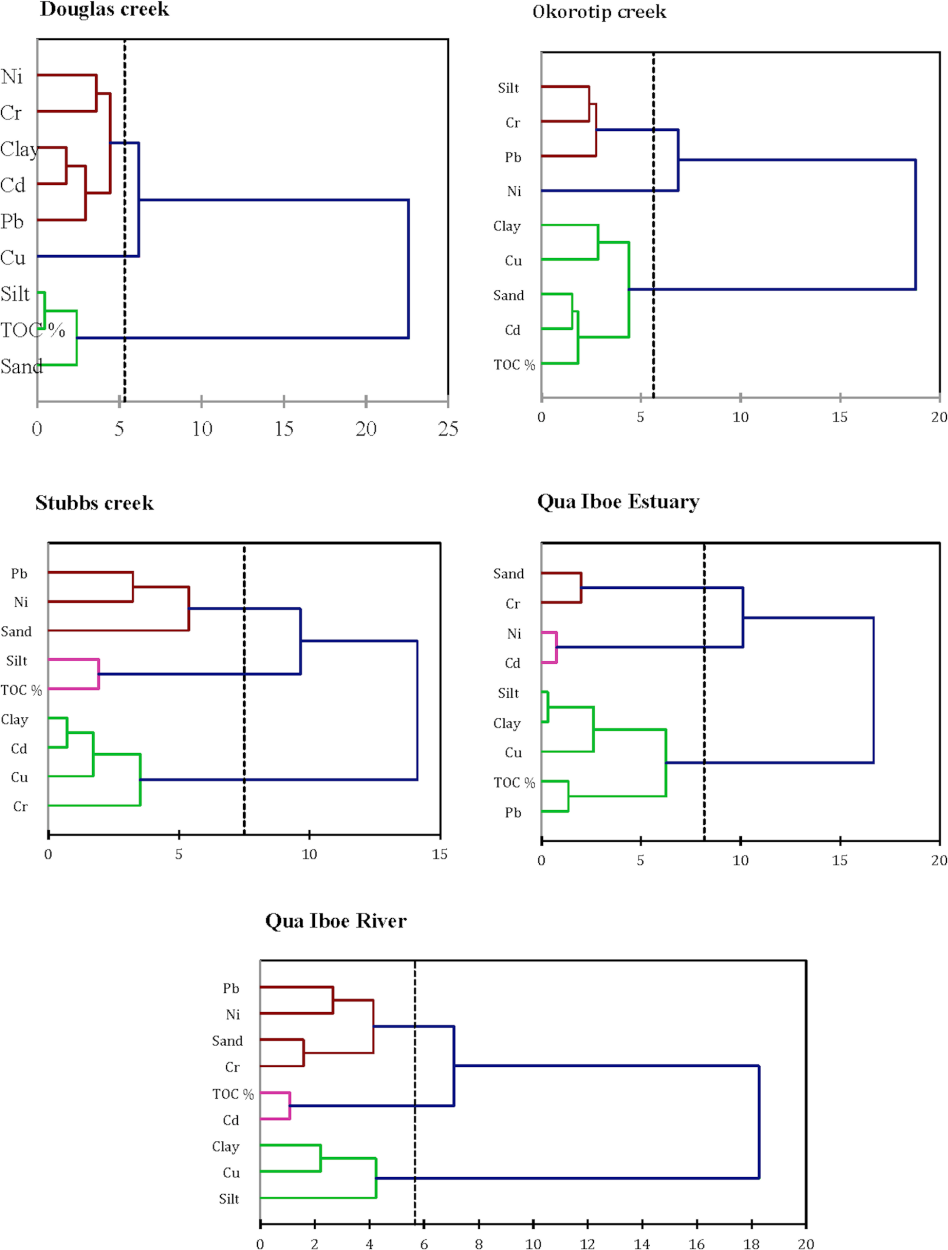
Hierarchical clustering analysis (Ward's Method) showing the relevant association among the parameters. Distance metrics are based on the Euclidean distance single linkage method (proximity matrix).

The dendrograms produced for each of the studied ecosystems are shown in Fig 7. The AHC grouped the metals, silt, clay, sand and TOC into two clusters on the basis of dissimilarity between different groups and similarity within a group. Cluster 1 comprises Cd, Cr, Cu, Ni, Pb and clay; Cr, Ni, Pb and silt; Ni, Pb, sand, silt and TOC; Cd, Cr, Ni and sand; and Cr, Ni, Pb and sand for the DOU, OKT, STB, QUE and QUR sites, respectively. It is shown that these trace metals are primarily clustered with sediment constituents (sand, silt, clay and TOC). This further highlights the vital role played by sediment in the transport, deposition, remobilization and accumulation of these metals in their respective aquatic ecosystems. However, at the DOU site, all investigated trace elements except Cu appear to have a common anthropogenic origin. On the other hand, cluster 2 comprises Cd and Cu for OKT site; Cd, Cr and Cu for STB site; Cu and Pb for QUE site; and Cu for QUR site. Moreover, the investigated metals appeared to indicate independent concentration variations in relation to the different aquatic ecosystems. In general, it is worth noting that certain trace metals were characteristically indicated in both clusters, highlighting the fact that metals distributions were distinctively varied in all studied ecosystems, and also that trace metal contamination could have emanated from multiple emission sources.

### 3.3 Contamination assessment

**Fig 8:**
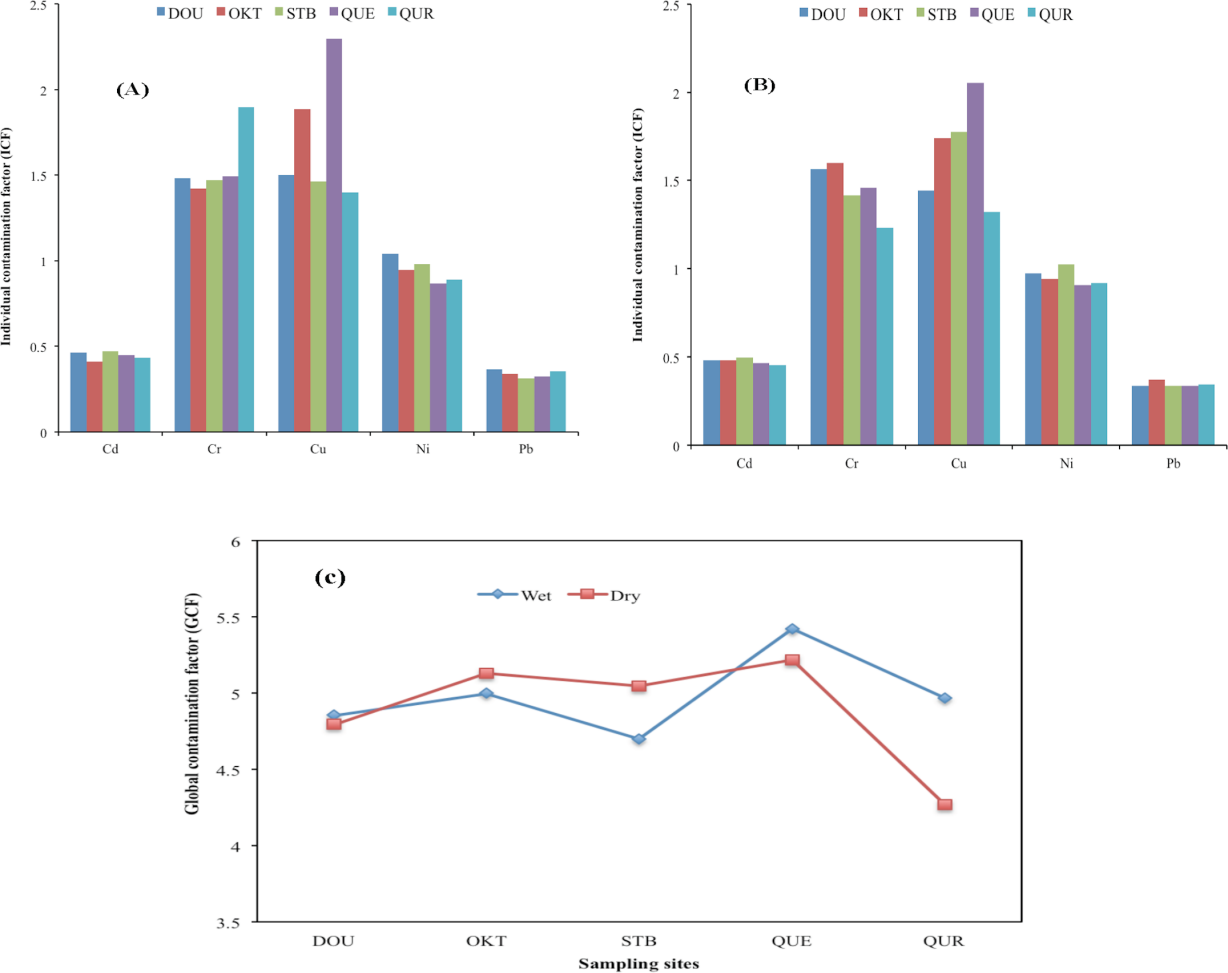
Weighted individual contamination factors (ICF) for wet (a) and dry (b) seasons and the global contamination factor for studied sites (c).

The degree of ecological risk associated with trace metals could be related to their retention times by determining corresponding contamination factors. According to [49], a high contamination factor of the metals implies low retention time and high degree of risk to the aquatic environment. In this study, the individual contamination factor was calculated and employed to estimate the relative retention time of benthic sedimentary metals. The individual contamination factors of each metal in the benthic sediment samples at the various investigated sites during the wet and dry seasons are presented in Fig 8A and 8B. The global contamination factor is shown in Fig 8C.

The highest and lowest levels of ICF for Cd were obtained at DOU and OKT sites, respectively, with corresponding ICF values of 0.511 and 0.312. The highest level of ICF for Cr was recorded at OKT site with a value of 1.696, while the lowest level was obtained at QUR site with a value of 0.603. Cu had the highest (2.643) and lowest (1.249) ICF levels at QUE and QUR study sites, respectively, with mean levels of 1.709 ±0.438 and 1.667 ±0.306 for the wet and dry seasons, respectively. The highest level of ICF (1.098) for Ni was computed for the STB site, while the lowest level (0.777) was obtained at QUR, with mean values of 0.943 ±0.107 and 0.954 ±0.069 calculated for all sites during the wet and dry seasons, respectively. Results indicated that the ICF levels for Pb were relatively low, implying that the retention times for Pb were comparatively high. The highest ICF level for Pb was obtained at OKT site with a value of 0.439, while the lowest ICF level was recorded at QUE, with a value of 0.266. The mean levels computed for all sites were 0.338 ±0.033 and 0.343 ±0.031 for the wet and dry seasons, respectively.

The individual contamination factor results showed relatively high levels for Cr and Cu (Fig 8), indicating the high potential mobility and bioavailability for these trace metals, whereas Cd and Pb had the lowest. The ICF computed for Ni also showed significant potential to be released from sediment into the overlying water column in the mangrove ecosystems. More so, the ICF contamination trend in the benthic sediments at all studied sites was Cu>Cr>Ni>Cd>Pb. However, the weighted concentration effects of Cu, Cr and Ni with comparatively moderate contamination factors imply enhanced ecological risks from these metals to the aquatic biota.

The GCF values for all investigated ecosystems ranged between 5.424 and 4.269 for both wet and dry seasons. These results indicated that the benthic sediments were moderately (6 < GCF < 12) impacted by trace metals. However, enhanced anthropogenic activities in this region may likely lead to heightened degree of contamination, which could result in considerable risks to the aquatic ecosystems, local floras and faunas.

**Fig 9:**
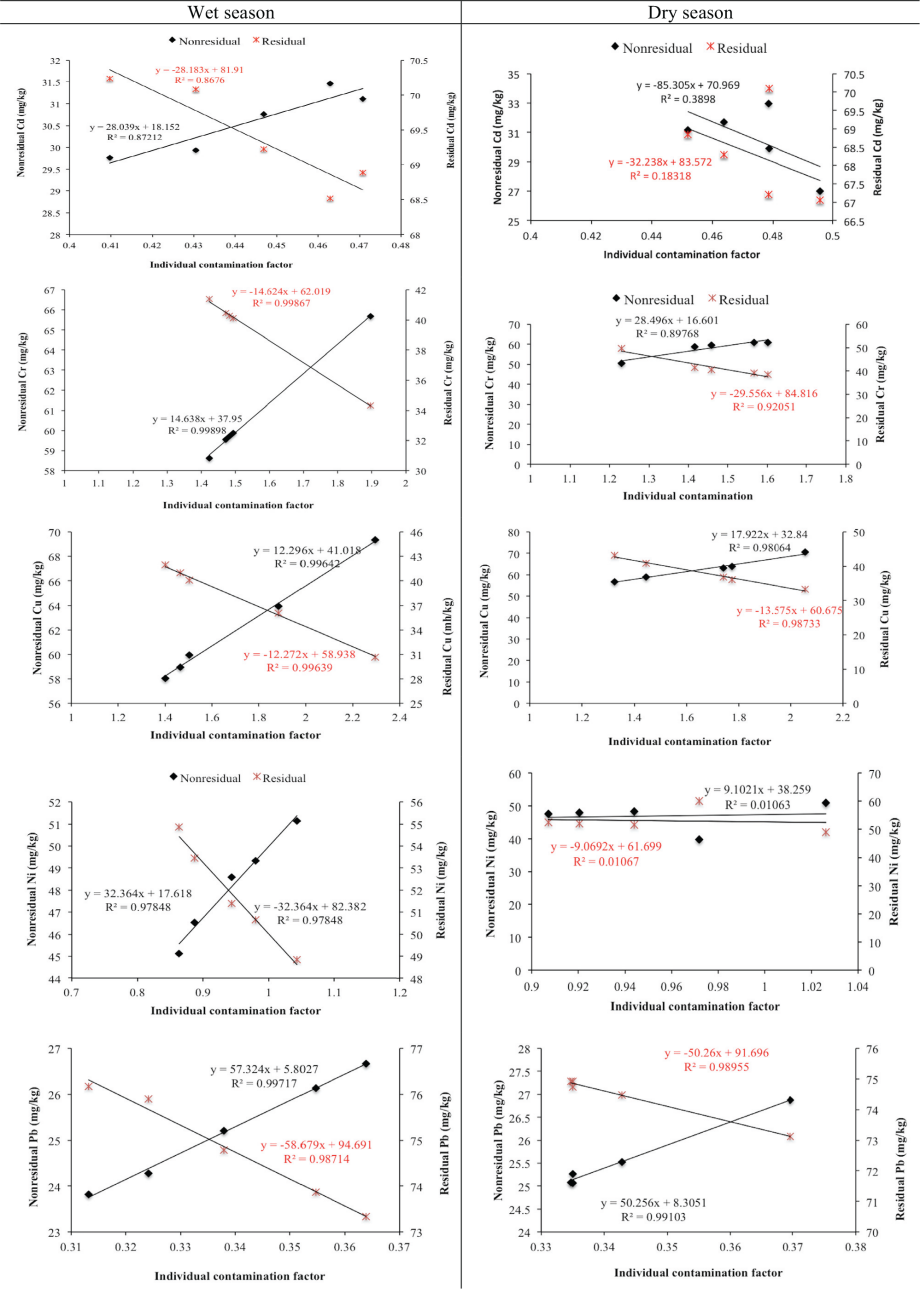
Relationships between trace metals in nonresidual/residual geochemical fractions and their corresponding individual contamination factors (ICFs) during the wet and dry seasons. The regression lines and equations in black color indicate metals in nonresidual fractions and ICFs; the regression lines and equations in red color represent metals in residual fractions and ICFs.

Chemical fractionation is an important technique that provides distinctive information about the origin of trace metals. Trace metals in the nonresidual fractions (exchangeable + carbonates bound + reducible + oxidizable) are an indication of anthropogenic influences, while those in residual geochemical phases can be attributed to lithogenic sources [53–55]. The relationships between the studied trace metals (Cd, Cr, Cu, Ni and Pb) in nonresidual/residual fractions and associated individual contamination factors in benthic sediments of the investigated estuarine and freshwater ecosystems are presented in Fig 9. As indicated in the linear plots, there was a significant and positive correlations between the nonresidual fractions and the individual contamination factors of Cd, Cr, Cu, Ni and Pb during the wet season, while a strong relationship was established between the nonresidual fractions and the correlative ICF values of Cr, Cu and Pb during the dry season. Cd and Ni showed very weak correlations. The linear relationships between residual fractions and the corresponding ICFs of metals revealed comparable negative but strong correlations for Cd, Cu and Pb, while weak linear relationships were observed for Cd and Ni.

## 4.0 Conclusion

In the present study, the occurrence, contamination, sources and associated risks of Cd, Cr, Cu, Ni and Pb in benthic sediments from five equatorial freshwater, riverine and estuarine ecosystems were investigated. Multivariate statistical approaches including principal component analysis, correlation test and agglomerative hierarchical cluster analysis were employed in characterizing the degree of contamination, sources and interrelationships of trace metals. Useful indices, guidelines and quotients were also used in ascertaining the extent of sediment contamination by individual metals. The ecological risk index by individual contamination factor shows significant potential mobility and bioavailability for Cu, Cu and Ni. Global contamination factor identifies the ecosystems as moderate pollution hotspots. However, enhanced degree of contamination due to anthropogenic activities in the region could result in considerable risks to these aquatic systems and biotas. The principal component and agglomerative clustering analyses indicate that trace metal contaminations in the ecosystems were influenced by multiple pollution sources. Results also indicate that multistep speciation of sediments provides a more accurate evaluation of mobility, bioavailability and risk of metals in aquatic ecosystems. The result of this study will provide valuable baseline information for future contamination and risk assessments of these littoral ecosystems.

## References

[1] Benson NU, Udosen ED, Akpabio O. Interseasonal distribution and partitioning of heavy metals in subtidal sediment of Qua Iboe Estuary and associated Creeks, Niger Delta (Nigeria). 2008; Environ. Monitor. Assessment 146(1–3): 253–265. 10.1007/s10661-007-0077-5 18066674

[2] Essien JP, Benson NU, Antai SP. Seasonal dynamics of physicochemical properties and heavy metal burdens in Mangrove sediments and surface water of the brackish Qua Iboe Estuary, Nigeria. 2008; Toxicological and Environmental Chemistry. 90(2): 259–273. 10.1080/02772240701550497

[3] Essien JP, Essien V, Olajire AA. Heavy metal burdens in patches of asphyxiated swamp areas within the Qua Iboe estuary mangrove ecosystem. 2009; Environmental Research, 109, 690–696. 10.1016/j.envres.2009.04.005 19464676

[4] Udosen ED, Benson NU. Spatio-temporal distributions of heavy metals in sediments and surface water in Stubbs Creek, Nigeria. 2006; Trends in Applied Sciences and Research, 1(3): 292–300. 10.3923/tasr.2006.292.300

[5] Essien JP, Antai SP. Negative direct effects of oil spill on beach microalgae in Nigeria. 2005; World Journal of Microbiology and Biotechnology, 21(4): 567–573.

[6] Asuquo FE. Tarballs in Ibeno-Okposo beach of South Eastern Nigeria. 1991; Marine Pollution Bulletin, 22: 150–151.

[7] Benson NU, Essien JP, Ebong GA, Williams AB. Petroleum hydrocarbons and limiting nutrients in *Macura reptantia*, *Procambarus clarkii* and benthic sediment from Qua Iboe Estuary, Nigeria. 2008; The Environmentalist, 28(3): 275–282.

[8] Benson NU, Essien JP, Williams AB, Bassey DE. Mercury accumulation in fishes from tropical aquatic ecosystems in the Niger Delta of Nigeria. 2007; Current Science. 96 (2): 781–785.

[9] Maanan M, Landesman C, Maanan M, Zourarah B, Fattal P, Sahabi M. Evaluation of the anthropogenic influx of metal and metalloid contaminants into the Moulay Bousselham lagoon Morocco, using chemometric methods coupled to geographical information systems. 2015; Environ. Sci. Pollut. Res. 20: 4729–4741. 10.1007/s11356-012-1399-6 23292198

[10] Goher ME, Farhat HI, Abdo MH, Salem SG. Metal pollution assessment in the surface sediment of Lake Nasser, Egypt. 2014; Egyptian Journal of Aquatic Research 40: 213–224.

[11] Benson NU, Etesin MU. Metal contamination of surface water, sediment and *Tympanotonus fuscatus var radula* of Iko River and environmental impact due to Utapete gas flare station, Nigeria. 2008; Environmentalist. 28(3): 195–202. 10.1007/s10669-007-9127-3

[12] Nguyen HL, Braun M, Szaloki I, Baeyens W, Van Grieken R, Leermakers M. Tracing the metal pollution history of the Tisza River through the analysis of a sediment depth profile. 2009; Water Air Soil Pollution. 200: 119–132.

[13] Díaz-de Alba M, Galindo-Riano MD, Casanueva-Marenco MJ, García-Vargas M, Kosore CM. Assessment of the metal pollution, potential toxicity and speciation of sediment from Algeciras Bay (South of Spain) using chemometric tools. 2011; J. Hazard. Mater. 190: 177–187. 10.1016/j.jhazmat.2011.03.020 21470776

[14] Lin YC, Chang-Chien GP, Chiang PC, Chen WH, Lin YC. Multivariate analysis of heavy metal contaminations in seawater and sediments from a heavily industrialized harbor in Southern Taiwan. 2013; Marine Pollution Bulletin. 76(1–2): 266–275. 10.1016/j.marpolbul.2013.08.027 24054783

[15] Carman CM, Li XD, Zhang G, Wai OWH, Li YS. Trace metal distribution in sediments of the Pearl River Estuary and the surrounding coastal area, South China. Environ. Pollution. 147, 311–323.Burton, G. A. Jr., 2002. Sediment quality criteria in use around the world. 2007; Limnol. 3: 65–75 10.1016/j.envpol.2006.06.028 17000039

[16] Ana TL, Paula T, Salomé FPA, João XM, Eduardo FDS. Environmental impact of mining activities in the Lousal area (Portugal): chemical and diatom characterization of metal contaminated stream sediments and surface water of Corona stream. 2011; Sci. Total Environ 409: 4312–4325. 10.1016/j.scitotenv.2011.06.052 21802708

[17] Hossein P, Zahra A, Parvin F, Mahmood K, Nematullah K, Abdolreza K. Bioavailability and concentration of heavy metals in the sediments and leaves of grey mangrove, Avicennia marina (Forsk.) Vierh, in Sirik Azini Creek, Iran. 2011; Biol Trace Elem Res 143:1121–1130. 10.1007/s12011-010-8891-y 21053092

[18] Gibbs RJ. Metals of the bottom muds in Townsville harbour, Australia. 1993; Environmental Pollution. 81: 297–300. 15091815 10.1016/0269-7491(93)90212-7

[19] Sahuquillo A, Rigol A, Rauret G. Overview of the use of leaching/extraction tests for risk assessment of trace metals in contaminated soils and sediments. 2003; TrAC Trends Anal. Chem. 22 (3): 152–159.

[20] Förstner U, Salomons W. Trends and challenges in sediment research the role of sediments in river basin management. 2008; 8, 281–283.

[21] Morelli G, Gasparon M. Metal Contamination of Estuarine Intertidal Sediments of Moreton Bay, Australia. 2014; Marine Pollution Bulletin. 89(1–2): 435–43. 10.1016/j.marpolbul.2014.10.002 25457811

[22] Dong A, Zhai S, Zabel M, Yu Z, Zhang H, Liu F. Heavy metals in Changjiang estuarine and offshore sediments: responding to human activities. 2012; Acta Oceanol. Sin. 31: 88–101.

[23] Wang J, Liu R, Zhang P, Yu W, Shen Z, Feng C. Spatial variation, environmental assessment and source identification of heavy metals in sediments of the Yangtze River Estuary. 2014; Marine Pollution Bulletin 87, 364–373. 10.1016/j.marpolbul.2014.07.048 25103899

[24] Cheng Z, Man YB, Nie XP, Wong MH. Trophic relationships and health risk assessments of trace metals in the aquaculture pond ecosystem of Pearl River Delta, China. 2013; Chemosphere 90, 2142–2148. 10.1016/j.chemosphere.2012.11.017 23219406

[25] Liu WX, Li XD, Shen ZG, Wang DC, Wai OWH, Li YS. Multivariate statistical study of heavy metal enrichment in sediments of the Pearl River Estuary. 2003; Environmental Pollution 121(3): 377–388. 12685766 10.1016/s0269-7491(02)00234-8

[26] Loska K, Wiechula D. Application of principal component analysis for the estimation of source of heavy metal contamination in surface sediments from the Rybnik Reservoir. 2003; Chemosphere 51:723–733. 12668031 10.1016/S0045-6535(03)00187-5

[27] Han YM, Du PX, Cao JJ, Posmentier ES. Multivariate analysis of heavy metal contamination in urban dusts of Xi’an, Central China. 2006; Sci. Total Environ. 355:176–186 15885748 10.1016/j.scitotenv.2005.02.026

[28] Zhang C, Fay D, McGrath D, Grennan E, Carton OT. Statistical analyses of geochemical variables in soils of Ireland. 2008; Geoderma 146: 378–390.

[29] Zhang HG, Cui BS, Zhang KJ. Heavy metal distribution of natural and reclaimed tidal riparian wetlands in south estuary, China. 2011; J Environ Sci 23:1937–1946 10.1016/s1001-0742(10)60644-4 22432322

[30] Singh KP, Malik A, Sinha S, Singh VK, Murthy RC. Estimation of source of heavy metal contamination in sediments of Gomti River (India) using principal component analysis. 2005; Water Air Soil Pollut. 166, 321–341.

[31] Li F, Zeng X-Y, Wu C-H, Duan Z-P, Wen Y-M, Huang G-R, et al Ecological Risks Assessment and Pollution Source Identification of Trace Elements in Contaminated Sediments from the Pearl River Delta, China. 2013; Biol Trace Elem Res 155:301–313. 10.1007/s12011-013-9789-2 23975580 PMC3785707

[32] Jobson J.D. Applied Multivariate Data Analysis Volume II: Categorical and Multivariate Methods. Springer-Verlag, New York; 1992.

[33] Jolliffe IT. Principal Component Analysis, Second Edition. Springer, New York; 2002.

[34] Chang ML, Sun YC, Doong RA, Wu SC, Fu CT. Concentrations and correlations of trace metals in estuarine sediments-interpretation by multivariate statistical analysis and elemental normalization. 2007; J Environ Eng Manage 17:143–150

[35] Ianni C, Magi E, Soggia F, Rivaro P, Frache R. Trace metal speciation in coastal and off-shore sediments from Ross Sea (Antarctica). 2009; Microchemical Journal. 10.1016/j.microc.2009.07.016

[36] Passos EDA, Alves JC, dos Santos IS, Alves JDPH, Garcia CAB, Spinola Costa AC. Assessment of trace metals contamination in estuarine sediments using a sequential extraction technique and principal component analysis. 2010; Microchemical Journal, 96(1): 50–57.

[37] Radojevic M, Bashkin VN. Practical Environmental Analysis. Royal Society of Chemistry, 465pp; 1999

[38] Schumacher BA. Methods for the determination of total organic carbon (TOC) in soils and sediments. 2002; EPA/NCEA-C- 1282 EMASC-001, U.S. EPA, Las Vegas, NV.

[39] Folk RL. The petrology of sedimentary rocks: Austin, Texas, Hemphill Publishing Co., 182 p; 1974.

[40] AOAC. Methods for Soil Analysis,12^th^ Edition. Association of Official Analytical Chemist, Washington, D.C.; 1975.

[41] Tessier A, Campell P, Bison M. Sequential extraction procedure for the speciation of particulate trace metals. 1979; Analytical Chemistry, 51 (7): 844–850.

[42] Popek EP. Sampling and Analysis of Environmental Pollutants: A Complete Guide AcademicPress, USA, p.356; 2003.

[43] Ward JH. Hierarchical grouping to optimize an objective function. 1963; Journal of the American Statistical Association, 58: 238–244.

[44] Arabie P, Hubert LJ, De Soete G. Clustering and Classification. World Scientific, Singapore; 1996.

[45] Everitt BS, Landau S, Leese M. Cluster analysis (4th edition). Arnold, London; 2001.

[46] XLSTAT, 2015. XLSTAT Pro. Addinsoft SARL, Paris, France.

[47] Wang J, Liu R, Zhang P, Yu W, Shen Z, Feng C. Spatial variation, environmental assessment and source identification of heavy metals in sediments of the Yangtze River Estuary. 2014; Marine Pollution Bulletin 87: 364–373. 10.1016/j.marpolbul.2014.07.048 25103899

[48] Nemati K, Bakar NK, Abas MR. Investigation of heavy metals mobility in shrimp aquaculture sludge-Comparison of two sequential extraction procedures. 2009; Microchemical Journal, 91(2): 227–231.

[49] Saleem M, Iqbal J, Shah MH. Geochemical speciation, anthropogenic contamination, risk assessment and source identification of selected metals in freshwater sediments—A case study from Mangla Lake, Pakistan. 2015; Environmental Nanotechnology, Monitoring & Management, 1–10. 10.1016/j.enmm.2015.02.002

[50] Zhao S, Feng C, Yang Y, Niu J, Shen Z. Risk assessment of sedimentary metals in the Yangtze Estuary: new evidence of the relationships between two typical index methods. 2012; J. Hazard. Mater. 241–242: 164–172. 10.1016/j.jhazmat.2012.09.023 23083940

[51] Bastami KD, Bagheri H, Kheirabadi V, Zaferani GG, Teymori MB, Hamzehpoor A, et al Distribution and ecological risk assessment of heavy metals in surface sediments along southeast coast of the Caspian Sea. 2014; Marine Pollution Bulletin 81: 262–267. 10.1016/j.marpolbul.2014.01.029 24606766

[52] Kaiser HF. An index of factorial simplicity. 1974; Psychometrika, 39: 31–36.

[53] Gao X, Chen CTA. Heavy metal pollution status in surface sediments of the coastal Bohai Bay. 2012; Water Res. 46: 1901–1911. 10.1016/j.watres.2012.01.007 22285040

[54] Islam MS, Ahmed MK, Raknuzzaman M, Habibullah-Al-Mamun M, Islam MK. Heavy metal pollution in surface water and sediment: A preliminary assessment of an urban river in a developing country. 2015; Ecological Indicators, 48: 282–291.

[55] Zhuang W, Gao X. Integrated assessment of heavy metal pollution in the surface sediments of the Laizhou Bay and the coastal waters of the Zhangzi Island, China: Comparison among typical marine sediment quality indices. 2014; PLoS ONE 9(4): e94145 10.1371/journal.pone.0094145 24709993 PMC3978014

